# Correlations Between Different Angiogenic and Inflammatory Factors in Vitreous Fluid of Eyes With Proliferative Diabetic Retinopathy

**DOI:** 10.3389/fmed.2021.727407

**Published:** 2021-09-28

**Authors:** Guanrong Wu, Baoyi Liu, Qiaowei Wu, Changting Tang, Zijing Du, Ying Fang, Yijun Hu, Honghua Yu

**Affiliations:** ^1^School of Medicine, South China University of Technology, Guangzhou, China; ^2^Department of Ophthalmology, Guangdong Provincial People's Hospital, Guangdong Academy of Medical Sciences, Guangzhou, China; ^3^Aier Institute of Refractive Surgery, Refractive Surgery Center, Guangzhou Aier Eye Hospital, Guangzhou, China; ^4^Aier School of Ophthalmology, Central South University, Changsha, China

**Keywords:** diabetic retinopathy, proliferative diabetic retinopathy, angiogenesis, inflammation, vitreous fluid

## Abstract

**Purpose:** To investigate the expression of various angiogenesis and inflammation mediators in the vitreous fluid of eyes with proliferative diabetic retinopathy (PDR).

**Methods:** A total of 38 eyes with PDR and 37 control eyes were included. Vitreous fluid was collected during vitrectomy. Vitreous levels of colony stimulating factor-1 receptor (CSF-1R), syndecan-1, placental growth factor (PIGF), and angiopoietin-like protein 4 (ANGPTL-4) were measured by multiplex immunoassay. Vitreous levels of vascular endothelial growth factor (VEGF), interleukin-6 (IL-6), interleukin-8 (IL-8), monocyte chemotactic protein-1 (MCP-1), tumor necrosis factor-α (TNF-*α*), and intercellular adhesion molecule-1 (ICAM-1) were measured by cytometric beads array. Levels of these mediators were compared between the PDR and control eyes. Correlations between levels of different mediators and between these mediators and kidney function metrics in the PDR group were also analyzed.

**Results:** Vitreous levels of syndecan-1, PIGF, ANGPTL-4, VEGF, and IL-8 were significantly higher in the PDR group compared to the control group (all *p* < 0.05). Levels of VEGF were significantly correlated with levels of syndecan-1, PIGF, and ANGPTL-4 (*r* = 0.370 to 0.497, all *p* < 0.05). Significant positive correlations were detected between levels of any two of the following mediators including syndecan-1, PIGF, ANGPTL-4, and IL-8 (*r* = 0.370 to 0.906, all *p* < 0.05). Apart from VEGF, levels of these mediators were positively correlated with serum creatinine and blood urea nitrogen (*r* = 0.328 to 0.638, all *p* < 0.05), and negatively correlated with fasting blood glucose and estimated glomerular filtration rate (*r* = −0.325 to −0.603, all *p* < 0.05).

**Conclusions:** Correlations between different angiogenesis and inflammation mediators were observed in eyes with PDR, suggesting cross-talks of different angiogenesis and inflammation pathways in the pathogenesis of PDR. The levels of angiogenesis and inflammation in PDR are correlated with kidney damage, indicating possible common pathways in diabetic retinopathy and nephropathy.

## Introduction

Diabetic retinopathy (DR) is the most common cause of vision loss in diabetes mellitus (DM). The overall prevalence of any DR in the world is 34.6% and prevalence of vision-threatening DR is estimated to be 10.2% (1). The number of people affected by moderate to severe vision impairment due to DR is estimated to be 2.6 million (2). The prevalence of DR increases with increasing DM duration, hemoglobin A1c, and blood pressure (1). Proliferative diabetic retinopathy (PDR) is the most vision-threatening type of DR and its prevalence is estimated to be 6.96% worldwide (1). PDR patients are at high risks of severe vision loss due to complications such as tractional retinal detachment and vitreous hemorrhage, and exploration of the mechanisms of PDR is critical for the treatment and prevention of the disease.

Angiogenesis and inflammation are two important mechanisms for the development of DR, including PDR (3). It has been shown that vascular endothelial growth factor (VEGF) plays a critical role in the development of the disease and anti-VEGF therapy has been commonly used in patients with PDR (4). Other angiogenesis and inflammation mediators such as placental growth factor (PIGF), angiopoietin-like protein 4 (ANGPTL-4), interleukin-6 (IL-6), interleukin-8 (IL-8), monocyte chemotactic protein-1 (MCP-1), tumor necrosis factor-α (TNF-*α*), and intercellular adhesion molecule-1 (ICAM-1) are also suggested to be involved in the pathogenesis of PDR (5–8). Colony stimulating factor-1 (CSF-1) and its receptor (CSF-1R) have also been found to be involved in the development of PDR and retinal inflammation (9, 10). And expression of syndecan-1 has been found to be elevated in vitreous of eyes with PDR (11, 12). Treatment targeting these mediators may be potential replacement therapy for anti-VEGF treatment, which is limited by short duration of action and heterogeneity of efficacy.

In the present study, we aim to investigate the expression of various angiogenesis and inflammation mediators mentioned above in the vitreous fluid of eyes with PDR.

## Materials and Methods

### Subjects

In this prospective study, 38 patients (38 eyes) with PDR who underwent vitrectomy for vitreous hemorrhage, proliferative epiretinal membrane, or tractional retinal detachment were recruited form the Department of Ophthalmology at Guangdong Provincial People's Hospital (GPPH) between August 2017 to August 2020. A total of 37 non-DM patients (37 eyes) who underwent vitrectomy for idiopathic pre-retinal membranes (IPM), idiopathic macular holes (IMH), or rhegmatogenous retinal detachment (RRD) were included as the control group. The exclusion criteria were: (1) coexistence of other ocular conditions associated with inflammation (such as age-related macular degeneration, glaucoma, uveitis etc.), (2) history of ocular surgery or trauma, (3) previous anti-VEGF treatment, (4) history of severe systemic inflammatory diseases, primary kidney diseases, or any other kidney diseases that are cause other than DM secondarily. The study was approved by the Research Ethics Committee of the Guangdong Provincial People's Hospital (Number:2016232A), and it was in accordance with the tenets of the Declaration of Helsinki. Informed consent was obtained from all patients before recruitment.

All patients underwent complete ocular examinations and measurements of blood pressure, fasting blood glucose (FBG), glycated hemoglobin (HbA1c), serum creatinine (sCr), blood urea nitrogen (BUN), and estimated glomerular filtration rate (eGFR) before surgery. The eGFR was calculated according to the Chronic Kidney Disease Epidemiology Collaboration (CKD-EPI) equation (13). Vitreous fluids were collected during pars plana vitrectomy using the 23-gauge trocar and cannula system (Alcon Laboratories, Inc. Fort Worth, Tex. the USA). About 0.2–0.4 ml of vitreous humor was aspirated into a sterile syringe before intraocular infusion. The vitreous samples were centrifuged immediately at 2,500 rpm at 4°C for 10 min. The supernatants were aspirated and subsequently stored at −80°C.

Vitreous levels of colony stimulating factor-1 receptor (CSF-1R), syndecan-1, placental growth factor (PIGF), and angiopoietin-like protein 4 (ANGPTL-4) were measured by multiplex immunoassay (Luminex Human Magnetic Assay, R&D Systems, Minneapolis, MN). Vitreous levels of vascular endothelial growth factor (VEGF), interleukin-6 (IL-6), interleukin-8 (IL-8), monocyte chemotactic protein-1 (MCP-1), tumor necrosis factor-α (TNF-*α*), and intercellular adhesion molecule-1 (ICAM-1) were measured by cytometric beads array BD Bioscience, San Jose, CA, USA. All samples were measured once. The minimum and maximum detection values were 9.77–2,500 pg/ml for IL-6, IL-8, MCP-1, TNF-*α*, and VEGF, 35.32–25,750 pg/ml for 2.92–710 pg/ml for PIGF, 526.43–383,770 pg/ml for ANGPTL-4, 136.49–99,500 pg/ml for CSF-1R, 38.94–28,390 pg/ml for MCP-1, and 38.94–28,390 pg/ml for syndecan-1. The *R*^2^ was higher than 0.99 in all curves. When values were outside the detection limit of the kit, the detection threshold was calculated based on the standard curve (7, 14).

### Statistical Analysis

Statistical analysis was performed using SPSS software package version 26 (SPSS. Inc, Chicago, IL, USA). Qualitative variables were presented as numbers and percentages. Quantitative variables were presented as median (lower quartile, upper quartile). Mann-Whitney *U*-test was applied to compare the basic characteristics, blood test parameters and levels of the angiogenesis and inflammation mediators between the PDR group and the control group. Spearman's correlation test was used to analyze the associations between different mediators which were significantly different between the two groups, or between the mediators and clinical characteristics/blood test parameters. For all the tests, *p* < 0.05 was considered statistically significant.

## Results

### Basic Characteristics

A total of 37 eyes with PDR and 38 control eyes were included. Basic characteristics of the two groups are shown in Table 1. There were significant differences in FBG, HbA1c, sCr, BUN, and eGFR between the two groups (all *p* < 0.001).

**Table 1:** Basic characteristics of the subjects.

**Characteristics**	**PDR (*n* = 38)**	**Non-DM (*n* = 37)**	***p*-value**
Age, y	57.00 (49.00, 69.00)	59.00 (51.00, 64.75)	0.480
Male/female	9/29	16/21	0.072
Duration of DM, years	8.30 (5.00, 10.00)	N/A	N/A
Duration of DR, months	12.00 (5.25, 16.13)	N/A	N/A
SBP, mmHg	128.00 (120.25, 144.00)	127.00 (116.25,138.50)	0.347
DBP, mmHg	83.00 (68.00, 89.25)	78.00 (73.25, 86.75)	0.718
FBG, mmol/l	10.10 (7.90, 13.27)	6.15 (5.33, 7.08)	<0.001^*^
HbA1c, %	7.70 (7.58, 7.90)	5.95 (5.50, 6.10)	<0.001^*^
sCr, μmol/l	169.40 (85.15, 297.90)	76.68 (60.08, 86.01)	<0.001^*^
BUN, mmol/l	9.96 (7.38, 16.42)	5.20 (4.38, 6.06)	<0.001^*^
eGFR, ml/min/1.73 m^2^	34.83 (18.16, 69.16)	93.10 (79.27, 100.24)	<0.001^*^

*PDR, proliferative diabetic retinopathy; DM, diabetes mellitus; SBP, systolic blood pressure; DBP, diastolic blood pressure; FBG, fasting blood glucose; HbA1c, glycated hemoglobin; sCr, serum creatinine; BUN, blood urea nitrogen; eGFR, estimated glomerular filtration rate*.

* *Statistically significant (p-value < 0.05)*.

### Vitreous Levels of the Angiogenesis and Inflammation Mediators

Vitreous levels of the angiogenesis and inflammation mediators are shown in Table 2. Vitreous levels of syndecan-1 (*p* = 0.004), PIGF (*p* < 0.001), ANGPTL-4 (*p* < 0.001), VEGF (*p* = 0.001), and IL-8 (*p* = 0.038) were significantly higher in the PDR group compared to the control group. No significant differences were found in vitreous levels of CSF-1R (*p* = 0.695), IL-6 (*p* = 0.719), MCP-1 (*p* = 0.611), TNF-α (*p* = 0.354), and ICAM-1 (*p* = 0.080).

**Table 2:** Vitreous levels of the angiogenesis and inflammation mediators.

**Mediators**	**PDR (*n* = 38)**	**Non-DM (*n* = 37)**	***p*-value**
Syndecan-1 (pg/ml)	196.75 (106.86, 446.55)	108.26 (68.78,171.45)	0.004^*^
CSF-1R (pg/ml)	24352.98 (13319.23, 58648.69)	39665.10 (13459.57, 51903.36)	0.695
PIGF (pg/ml)	35.60 (3.49, 283.08)	0.81 (0.37, 1.29)	<0.001^*^
ANGPTL-4 (pg/ml)	112378.13 (31458.05, 387799.98)	26890.31 (8022.31, 54353.15)	<0.001^*^
VEGF (pg/ml)	22.14 (5.40, 204.52)	3.15 (2.38, 4.15)	0.001^*^
IL-6 (pg/ml)	45.32 (11.31, 360.45)	31.84 (13.12, 207.56)	0.719
IL-8 (pg/ml)	50.71 (19.84, 256.68)	23.69 (10.70, 49.14)	0.038^*^
MCP-1 (pg/ml)	1107.18 (610.26, 2084.73)	1315.25 (590.25, 2400.45)	0.611
TNF-α (pg/ml)	8.81 (6.68, 46.28)	7.92 (6.79, 9.83)	0.354
ICAM-1 (pg/ml)	1209.64 (318.50, 2075.00)	572.37 (224.68, 1215.11)	0.080

*PDR, proliferative diabetic retinopathy; DM, diabetes mellitus*.

* *Statistically significant (p value < 0.05)*.

### Correlations Between Vitreous Levels of Different Angiogenesis and Inflammation Mediators

Among the angiogenesis and inflammation mediators significantly different between the two groups, correlation coefficients between vitreous levels of any two mediators are shown in Table 3. Significant correlations were detected between levels of syndecan-1 and levels of PIGF (*r* = 0.813), ANGPTL-4 (*r* = 0.859), VEGF (*r* = 0.471), and IL-8 (*r* = 0.826), between levels of PIGF and levels of ANGPTL-4 (*r* = 0.906), VEGF (*r* = 0.497), and IL-8 (*r* = 0.879), and between levels of ANGPTL-4 and levels of VEGF (*r* = 0.370) and IL-8 (*r* = 0.902) (all *p* < 0.05). No significant correlation was found between levels of VEGF and levels of IL-8 (*p* = 0.078).

**Table 3:** Correlations between vitreous levels of different angiogenesis and inflammation mediators in eyes with PDR.

**Mediators**	**Syndecan-1 (pg/ml)**	**PIGF (pg/ml)**	**ANGPTL-4 (pg/ml)**	**VEGF (pg/ml)**	**IL-8 (pg/ml)**
	***r* value**	***r* value**	***r* value**	***r* value**	***r* value**
	**(*p*-value)**	**(*p*-value)**	**(*p*-value)**	**(*p*-value)**	**(*p*-value)**
Syndecan-1 (pg/ml)		0.813 (<0.001^*^)	0.859 (<0.001^*^)	0.471 (0.003^*^)	0.826 (<0.001^*^)
PIGF (pg/ml)			0.906 (<0.001^*^)	0.497 (0.001^*^)	0.879 (<0.001^*^)
ANGPTL-4 (pg/ml)				0.370 (0.022^*^)	0.902 (<0.001^*^)
VEGF (pg/ml)					0.289 (0.078)

* *Statistically significant (p value < 0.05)*.

### Correlations Between Vitreous Levels of the Angiogenesis and Inflammation Mediators and Kidney Function Metrics

Among the mediators significantly different between the two groups, correlation coefficients between vitreous levels of the mediators and systemic factors are shown in Table 4. Significant positive correlations were found between sCr and levels of syndecan-1, PIGF, ANGPTL-4, and IL-8 (*r* = 0.328 to 0.572, all *p* < 0.05). Similar correlations were detected between BUN and levels of syndecan-1, PIGF, ANGPTL-4, and IL-8 (*r* = 0.447 to 0.638, all *p* < 0.05). The eGFR and FBG were found to be significantly negatively correlated with levels of syndecan-1, PIGF, ANGPTL-4, and IL-8 (*r* = −0.325 to −0.603, all *p* < 0.05).

**Table 4:** Correlations between vitreous levels of the angiogenesis and inflammation mediators and systemic factors.

**Systemic factors**	**FBG, mmol/l**	**HbA1c, %**	**sCr, μmol/l**	**BUN, mmol/l**	**eGFR, ml/min/1.73 m^2^**
	***r* value**	***r* value**	***r* value**	***r* value**	***r* value**
	**(*p*-value)**	**(*p*-value)**	**(*p*-value)**	**(*p*-value)**	**(*p*-value)**
Syndecan-1 (pg/ml)	−0.325 (0.047^*^)	−0.054 (0.746)	0.572 (<0.001^*^)	0.629 (<0.001^*^)	−0.603 (<0.001^*^)
PIGF (pg/ml)	−0.350 (0.031^*^)	−0.231 (0.163)	0.338 (0.038^*^)	0.447 (0.005^*^)	−0.356 (0.028^*^)
ANGPTL4 (pg/ml)	−0.362 (0.026^*^)	−0.279 (0.101)	0.428 (0.007^*^)	0.638 (<0.001^*^)	−0.453 (0.004^*^)
VEGF (pg/ml)	−0.168 (0.315)	−0.268 (0.104)	0.000 (0.998)	0.184 (0.270)	−0.019 (0.910)
IL-8 (pg/ml)	−0.403 (0.012^*^)	−0.226 (0.173)	0.328 (0.044^*^)	0.509 (0.001^*^)	−0.365 (0.024^*^)

*FBG, fasting blood glucose; HbA1c, glycated hemoglobin; sCr, serum creatinine; BUN, blood urea nitrogen; eGFR, estimated glomerular filtration rate*.

* *Statistically significant (p value < 0.05)*.

## Discussion

Angiogenesis and inflammation are the key feature of PDR and also thought to be critical mechanisms for PDR initiation and progression (3, 15). In this study, we showed higher vitreous levels of angiogenesis and inflammation mediators including syndecan-1, PIGF, ANGPTL-4, VEGF and IL-8 in eyes with PDR. We also detected significant positive correlations between vitreous levels of these mediators. Apart from VEGF, levels of these mediators were significantly correlated with FBG, sCr, BUN, and eGFR. Our results indicate that interactions between different angiogenesis and inflammation mediators are involved in the pathogenesis of PDR.

Results in our study showed that the vitreous levels of angiogenesis and inflammation mediators including syndecan-1, PIGF, ANGPTL-4, VEGF and IL-8 in the PDR group were significantly higher than in control group. Under high glucose (HG) and hypoxia conditions, increased hypoxia-inducible factors (HIFs) in the retina activated the transcription of multiple genes encoding angiogenesis mediators, including ANGPTL-4, VEGF, and PIGF (5, 16, 17). Elevated vitreous level of syndecan-1 was also detected in eyes of PDR in previous studies (11, 12). The reason for the increase of syndecan-1 might be due to activation of the heparinase/syndecan-1 axis or matrix metallopeptidases (MMPs)/syndecan-1 axis in retinal microvascular endothelial cells induced by HG, hypoxia, or inflammation (11, 12). On the other hand, HIFs can also lead to the up-regulation of chemokines, including IL-8, which can promote the infiltration of inflammatory cells (18). However, increased vitreous levels of several inflammation mediators noted in previous studies, including TNF-*α*, MCP-1, CSF-1R, ICAM-1 and IL-6, were not observed in our study. Similar negative findings were also noticed in some other studies (19, 20). The discrepancy might be due to different PDR phenotypes in different studies (21–23). Taken together, the roles of the angiogenesis and inflammation mediators in PDR are complicated, and the vitreous levels of these mediators may be affected by the phenotypes of PDR.

**Figure 1:**
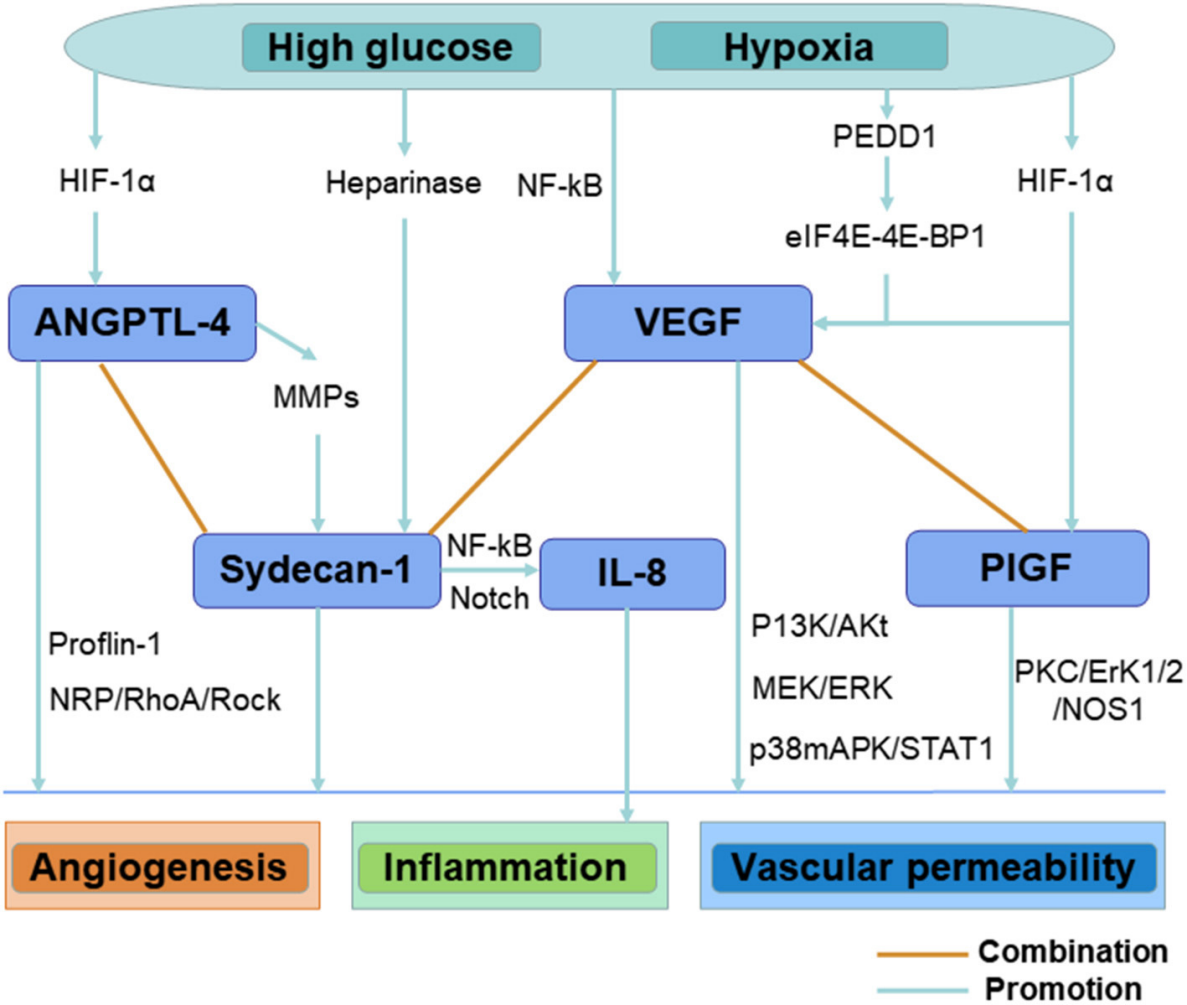
Illustration shows the interactions between different angiogenesis and inflammation mediators in eyes with PDR.

Correlation analysis revealed the complex relationships between different angiogenesis and inflammation mediators. Results in our study showed that the correlations between any two of following mediators including syndecan-1, PIGF, ANGPTL-4, and IL-8 were nearly two times stronger than those between VEGF and other mediators. These results suggested a critical role of the regulation of non-VEGF pathways in the pathogenesis of PDR. Emerging evidences suggest that ANGPTL4 may be a candidate target in DME and PDR treatment (5, 24). ANGPTL4 is up-regulated in hypoxic retinal Müller cells and plays an upstream role of VEGF, and it may accelerate the progression of PDR by increasing retinal angiogenesis and promoting vascular permeability (25, 26). A previous study demonstrated that inhibition of ANGPTL-4 was more efficient than inhibition of VEGF alone in reducing the angiogenesis effect in PDR, indicating pathways in addition to VEGF (25). What is more, ANGPTL-4 can induce the expression of MMPs, enhancing the shedding of syndecan-1 from the cell surface, and syndecan-1 is one of the ANGPTL-4 receptors and mediates ANGPTL-4-induced intracellular signaling (24, 27). Previous studies also reported that syndecan-1 could up-regulate the expressions of inflammation mediators such as IL-8 in chronic inflammation, leading to leukocyte chemotaxis and initiation of inflammatory response (28, 29). Therefore, the ANGPTL-4/MMP/syndecan-1/IL-8 axis may contribute to PDR progression (Figure 1). However, the interactions between different angiogenesis and inflammation mediators in PDR have not been completely elucidated, and future investigations are needed to explore these complicated mechanisms. Targeting both VEGF and non-VEGF angiogenesis pathways may be necessary for effective treatment or prevention of PDR. These findings suggest the value of non-VEGF angiogenesis pathways as the potential therapeutic targets of PDR.

We also revealed that levels of the angiogenesis and inflammation mediators were significantly correlated with indicators of kidney damage including sCr, BUN, and eGFR. These results suggested that kidney damage tended to be more severe in patients with higher levels of angiogenesis and inflammation in the eye. There are substantial evidences supporting the association between DR and diabetic nephropathy (DN) (30–35): (1) both DR and DN are characterized by diabetic microangiopathy; (2) there are common risk factors for the occurrence and progression of DR and DN; (3) the severity of DR is correlated with the degree of kidney damage; (4) multiple pathways have been considered to be involved in the development and progression of both DR and DN. On the one hand, elevated sCr and BUN and reduced eGFR in our study reflected microvascular damage of the kidney in the PDR patients. On the other hand, levels of the vitreous mediators were indicators of angiogenesis and inflammation in the retina of PDR. In our study, apart from VEGF, these angiogenesis and inflammation mediators were significantly correlated with kidney damage, consistent with previous studies showing significant correlations between serum levels of these mediators and renal functions (36–38), suggesting that non-VEGF pathways might be involved in the pathogenesis of both DR and DN. Increased serum levels of ANGPTL-4, syndecan-1, PIGF, and IL-8 were observed in diabetes patients with complications (26, 39, 40). It was shown that ANGPTL-4 expression was positively correlated with the amount of 24-h urine protein, sCr and the kidney weight index (41). It was also demonstrated that ANGPTL-4 was over-expressed in DN rat and associated with HG-induced cell proliferation, inflammatory response, and extracellular matrix accumulation in glomerular mesangial cells, mediating damage to the glomerular filtration barrier (41, 42). Besides, syndecan-1 shedding may also play a role in the development of DN (40). These evidences support that DR and DN might share a common pathogenesis and clinical course, indicating that therapy targeting non-VEGF pathways may be beneficial to hinder the progression of both DR and DN.

In addition, results in our study showed that angiogenesis and inflammation mediators were negatively correlated with FBG. These results might be due to the increased intensity of glycemic control in PDR patients.

There are several limitations of this study. Firstly, we did not detect the levels of mediators in NPDR patients or DM patients without DR, so we were unable to investigate the relationship between the vitreous levels of the angiogenesis and inflammation mediators with the severity of DR. We also did not detect the serum levels of these mediators. Therefore, we could not analyze the correlations between serum levels of these mediators and renal functions. Secondly, heterogeneity of the control group might change the profile of the angiogenesis and inflammation mediators in the vitreous fluid, masking the true effects of certain mediators in PDR. Thirdly, since our study was based on the human level, the exact mechanisms of the correlations between these mediators and cross-talks of angiogenic and inflammatory pathways in the pathogenesis of PDR require further investigation in animal experiments. Finally, population-based randomized studies with larger samples and basic research are needed to verify our results, as well as explore the regulation mechanisms of the angiogenesis and inflammation mediators in PDR.

## Conclusions

The present study revealed elevated vitreous levels of various angiogenesis and inflammation mediators and significant correlations between these mediators in eyes of PDR, providing clues to understand the link between angiogenesis and inflammation in PDR. Moreover, significant correlations between the vitreous levels of these mediators and kidney damage indicated a potential relationship between retinopathy and nephropathy in PDR patients.

## Data Availability Statement

The data analyzed in this study is subject to the following licenses/restrictions: The data used during the current study are available from the corresponding authors on reasonable request. Requests to access these datasets should be directed to Yijun Hu, huyijun2014@163.com.

## Ethics Statement

The studies involving human participants were reviewed and approved by Research Ethics Committee of the Guangdong Provincial People's Hospital. The patients/participants provided their written informed consent to participate in this study.

## Author Contributions

YH, HY, GW, and BL: conception and design. BL, QW, ZD, and YF: data collection and collation. GW and ZD: laboratory analysis. GW, BL, QW, and CT: data analysis and interpretation. YH and GW: manuscript writing. YH and HY: data interpretation and final review of the manuscript. All authors revised and approved the submitted manuscript.

## Funding

This work was supported by Grant 81870663 and 82171075 from the National Natural Science Foundation of China (HY), Grant KJ012019087 of the Outstanding Young Talent Trainee Program of Guangdong Provincial People's Hospital (HY), Grant KJ012019457 from the GDPH Scientific Research Funds for Leading Medical Talents and Distinguished Young Scholars in Guangdong Province (HY), Grant Y012018145 from the Talent Introduction Fund of Guangdong Provincial People's Hospital (HY), Grant A2021378 from the Medical Scientific Research Foundation of Guangdong Province, China (YH), Grant 2018SK50106 from the Technology Innovation Guidance Program of Hunan Province (YH), Grant AM1909D2 and AR1909D2 from the Science Research Foundation of Aier Eye Hospital Group (YH).

## Conflict of Interest

The authors declare that the research was conducted in the absence of any commercial or financial relationships that could be construed as a potential conflict of interest.

## Publisher's Note

All claims expressed in this article are solely those of the authors and do not necessarily represent those of their affiliated organizations, or those of the publisher, the editors and the reviewers. Any product that may be evaluated in this article, or claim that may be made by its manufacturer, is not guaranteed or endorsed by the publisher.
